# Seroprevalence of hepatitis e virus among primary school children

**DOI:** 10.12669/pjms.292.2821

**Published:** 2013-04

**Authors:** Nural Cevahir, Melek Demir, Ali Ihsan Bozkurt, Ahmet Ergin, Ilknur Kaleli

**Affiliations:** 1 Nural Cevahir, Department of Medical Microbiology, School of Medicine, Pamukkale University, Denizli, Turkey.; 2 Melek Demir, Department of Medical Microbiology, School of Medicine, Pamukkale University, Denizli, Turkey.; 3 Ali Ihsan Bozkurt, Department of Public Health, School of Medicine, Pamukkale University, Denizli, Turkey.; 4 Ahmet Ergin, Department of Public Health, School of Medicine, Pamukkale University, Denizli, Turkey.; 5 Ilknur Kaleli, Department of Medical Microbiology, School of Medicine, Pamukkale University, Denizli, Turkey.

**Keywords:** Anti-HEV Ab, Rural area, Urban area, Seroprevalence, School children

## Abstract

***Objectives:*** To investigate the seroprevalence of anti-hepatitis E virus antibody among primary school children in the two different areas of Denizli, Turkey.

***Methodology*** *:* Anti-HEV antibodies were investigated in 185 primary school children (91 from rural areas and 94 from urban areas of Denizli). The children were divided into two age groups as seven-year old group and fourteen-year old group. Samples were tested for anti-HEV Ab by an enzyme-linked immunoassay.

***Results*** *:* A total of 23 primary school children were anti-HEV Ab positive, giving a prevalence of 12.4%. The seroprevalence rate was 13.1% in rural areas and 11.7% in urban areas. The difference in the seropositive rates was not statistically significant (p>0.05). Among 185 primary school children, Anti-HEV antibodies were positive 17 (18.1%) in seven-year old group, and 6 (6.6%) in fourteen-year old group. The difference in the seropositive rates was statistically significant (p<0.05).

***Conclusions*** *:* There was no association between the anti-HEV Ab and gender, socioeconomic level, parental educational level, rural or urban areas. Anti-HEV Ab seroprevalence was higher in seven-year old children than fourteen-year old children.

## INTRODUCTION

Hepatitis E, previously known as enterically transmitted non-A, non-B hepatitis, is an infectious viral disease with clinical and morphological features of acute hepatitis.^1^^-^^3^ The hepatitis E virus (HEV) is an RNA virus, which is thought to spread via the fecal-oral route; outbreaks of hepatitis E have been attributed to water contaminated with HEV. Hepatitis E is endemic in many subtropical and tropical areas. Over 50 outbreaks have been reported in Southeast and Central Asia, the Middle East, northern and western parts of Africa, and Mexico. In these areas, hepatitis E occurs both in epidemic and sporadic forms.^2^^,^^4^ Person-to-person transmission is uncommon. HEV infection is self-limited, and has no chronic sequel. The highest attack rate appears to be among individuals between 15 and 40 years of age. Hepatitis E is generally rare in some regions in developed countries, but seroprevalence in some of the regions in these areas is higher than expected, suggesting that infection is in fact more widespreaded in the world. However, clinical features and risk factors of sporadic hepatitis E have not been well described.^1^^-^^5^

This study has been conducted in Western Turkey to investigate the seroprevalence for anti-HEV antibody in primary school children.

## METHODOLOGY

This study was performed for evaluation of seroprevalence of hepatitis E among healthy primary school children in Denizli, Turkey. Before the study, a written informed consent from parents were obtained. We did not perform any additional procedure with patients, other than drawing blood. Anti-HEV antibodies were investigated in 185 primary school children (91 from rural areas and 94 from urban areas of Denizli). The children were divided into two age groups as seven-year old group and fourteen-year old group.

 A blood samples were collected from each subject, and serum specimens were kept at –70 ^0^C waiting laboratory examination. Serum samples were tested enzyme -immunoAssay for anti-HEV IgG&M antibodies (BLK 7-EVAB, BLK diagnostics, Badalona, Spain). The ELISA was performed according to the protocols provided by the manufacturer.

Investigation of risk factors included age, sex, resident area, socio-economical level and parent’s education.

***Statistical analysis*** *:* The chi-square test was performed to check out possible correlations between the seropositive anti-HEV antibodies and risk factors included age, sex, resident area, socio-economical level and parent’s education. A p value of less than 0.05 was considered significant. The statistical analysis was performed using SPSS version 16 for windows.

## RESULTS

The study group included 185 primary school children who were the seven years of age and fourteen years of age. None of the children had any history of icterus. A total of 91 primary school children (47 female and 44 male) in rural area and a total of 94 primary school children (45 female and 49 male) in urban area were included this study. As expected, socio-economic and parental education levels were low in rural areas as compared to urban areas.

A total of 23 primary school children (10 female and 13 male) were anti-HEV Ab positive, giving a prevalence of 12.4%. The seroprevalence rates were 13.1% in rural areas and 11.7% in urban areas. The difference in the seropositive rates was not statistically significant (p>0.05). In rural areas, the seroprevalence rates were 19.5% for seven-year age group, and 6.6% for fourteen-year age group. In urban areas, the seroprevalence rates were 16.6% for seven-year age group, and 6.5% for fourteen-year age group. Among 185 primary school children, Anti-HEV antibodies were positive 17 (18.1%) in seven-year age group, and 6 (6.6%) in fourteen-year age group. The difference in the seropositive rates was statistically significant (p<0.05). Table-I shows the seroprevalence of anti-HEV Ab among primary school children in Denizli by age and residential areas.

**Table-I:** Age Seroprevalence of Anti-HEV Ab in both areas

Age groups	*Rural Areas*		*Urban areas*		*Total*	
	NoTested (n)	+ (%)	NoTested (n)	+ (%)	NoTested (n)	+ (%)
7 years	46	9 (19.5)	48	8 (16.6)	94	17 (18.1)
14 years	45	3 (6.6)	46	3 (6.5)	91	6 (6.6)
Total	91	12 (13.1)	94	11 (11.7)	185	23 (12.4)

There was no association between the anti-HEV Ab and gender, socioeconomic level, parental educational level, per schools in which parameters were studied. The seroprevalence of the age group of 14 years was lower than the age group of 7 years.

## DISCUSSION

Hepatitis E virus (HEV) is a major cause of acute hepatitis in many developing countries. HEV is an important enterically transmitted human pathogen with a worldwide distribution. It can cause sporadic cases as well as large epidemics of acute hepatitis. Epidemics are primarily waterborne in areas where water supplies are contaminated with HEV of human origin.^6^ Outbreaks can generally be traced back to contaminated water sources. The occurrence and magnitude of outbreaks are strongly associated with the hygienic conditions and population density in areas with a constant shortage of clean water. HEV particles have been detected in sewage.^2^

The prevalence of anti-HEV in healthy subjects has been studied in various populations worldwide to measure the extent of exposure to HEV. It has been found that anti-HEV antibodies are present in persons living in all geographical areas. In disease-endemic areas of Asia and Africa, the prevalence rates among healthy populations are much higher than those in non-endemic areas. In most disease-endemic areas, anti-HEV has been detected in as many as 5% of children less than 10 years of age, and this ratio increases to 10-40% among adults older than 25 years of age.^7^^,^^8^ These findings suggest that HEV infection is infrequent among young children in developing countries.^8^ The age-specific incidence was highest in persons aged 15-39 years, lower among those older than 40 years, and lowest in children less than 14 years.^9^ The low rate of infection among children less than 14 years suggests a lower susceptibility, which is unusual for enterically transmitted viruses. In contrast, other studies have suggested a high susceptibility of children to HEV infection.^2^^,^^10^ In addition, 12-56% of sporadic acute viral hepatitis in children from rural Egypt, urban Sudan, Somalia, and India are due to HEV infection.^10^^-^^15^ In a report from India, anti-HEV antibodies were detected in more than 60% of children below the age of 5 years.^16^

In our country, several studies have been carried out to reveal the HEV prevalence in the communities of various regions. According to these studies, anti- HEV seropositivity ranged from 3% and 29%.^17^ The prevalence rates of HEV seropositivity differ greatly. However, in various regions of Turkey, especially in Southeast Anatolia seropositivity was higher, the average rate was 29%.^17^ In our country, in childhood, HEV seroprevalence ranged from 1.1% to 26%.^17^ In one study performed in Istanbul, Turkey, seroprevalence of hepatitis E among children who were between 6 month-old and 15 year-old were determined as 2.1%.^18^ In Colak et al’s^19^ study, no antibody to HEV was detected in preschool children, while the prevalence of anti-HEV IgG was 1.6% in children attending school in Antalya, Turkey. In Atabek et al’s^20^ study, the HEV antibody prevalence was found 6.8% in 7-12 year-old group, and 8.9% seroprevalence rate in the 13-18 year-old group. In our study, the HEV antibody prevalence was found 12.4% in the primary school children. The anti-HEV antibody seroprevalence was found to be higher in 7 year-old children (18,1%) than 14 year-old children (6.6%), the difference was statistically significant (p<0.05). This rate is higher than previous studies in Istanbul and in Antalya, Turkey. But, our rates were consistent with other regions of Turkey. The Denizli region is a developed and industrialised region in western Turkey, and urban infrastructure of sewage is much better than the eastern and southeast part of the country. The prevalence rate found in our study was similar to the results obtained in previous studies in the eastern and southeast part of the country. In our study the prevalence rates of anti-HEV positivity was similar to urban and rural area. Both urban and rural areas had poor quality water sources. Perhaps, in our city, the water supplies are not regularly chlorinated. The poor environmental, hygienic conditions, and poor sanitation may play a role in high anti-HEV seroprevalence. This suggests an influence of local factors and living conditions on HEV prevalence.

Serum Ig M, Ig A and IgG antibodies to HEV develop during the course of the infection. IgM anti-HEV appears first and is closely followed by IgG anti-HEV. Ig M anti-HEV disappears within a few months. Although IgG anti-HEV lasts longer, the duration has not been adequately determined. There is some evidence that antibody development to hepatitis E virus is not as long lasting as is antibody to hepatitis A virus. Many reports suggest that Ig G antibodies to HEV are relatively short-lived.^1^^,^^3^ Most studies indicate that Ig G anti-HEV titers peak approximately 4 weeks after infection and decline rapidly.^9^^,^^21^ The protective role of anti-HEV antibodies in human requires further study. The occurence of large hepatitis E epidemics among adults in disease-endemic areas suggests either that anti-HEV antibody may not be fully protective or that antibody levels decline with time and gradually reach an unprotective level.^8^^,^^9^^,^^22^ In our study, the seroprevalence of anti-HEV antibody was lower in 14 year-old age group than 7 year old age group. Perhaps, HEV infections occur during early childhood. Therefore, anti-HEV antibody levels decline with age, seroprevalence of anti-HEV antibody may be low in the fourteen-year old group. Additionally, although we cannot explain the reason for that, the reason might be the personal hygiene which is much better at the 14 year-olds. The strict personal hygiene might reduce the incidence of HEV. The data obtained indicate a high susceptibility of children for HEV infection.

Our data showed that seroprevalence of anti-HEV antibody is high among primary school children in our city and could be due to an insufficient sanitation in Denizli. In addition, HEV should be considered in all children with acute hepatitis.
